# Methaemoglobinaemia and the radical curative efficacy of 8-aminoquinoline antimalarials

**DOI:** 10.1111/bcp.15219

**Published:** 2022-01-27

**Authors:** Nicholas J White, James A Watson, J Kevin Baird

**Affiliations:** 1 Mahidol Oxford Research Unit, Faculty of Tropical Medicine, Mahidol University, Bangkok, Thailand; 2 Centre for Tropical Medicine and Global Health, Nuffield Department of Medicine, University of Oxford, Oxford, United Kingdom; 3 Eijkman-Oxford Clinical Research Unit, Eijkman Institute for Molecular Biology, Jakarta, Indonesia

**Keywords:** methaemoglobinaemia, *P. vivax*, 8-aminoquinolines, primaquine, tafenoquine

## Abstract

Methaemoglobin results from the oxidation of ferrous to ferric iron in the centre of the haem moeity of haemoglobin. The production of dose-dependent methaemoglobinaemia by 8-aminoquinoline antimalarial drugs appears to be associated with, but is not directly linked to, therapeutic efficacy against latent *Plasmodium vivax* and *P. ovale* malarias (radical cure). Iatrogenic methaemoglobinaemia may be a useful pharmacodynamic measure in 8-aminoquinoline drug and dose optimization.

## Introduction

Hepatic latency in vivax and ovale malarias (dormant liver stage parasites called hypnozoites) requires treatment with 8-aminoquinoline drugs in order to prevent recurrent attacks called relapses. This is called radical cure). The first 8-aminoquinoline (variously named plasmoquine, plasmochin, or pamaquine) was discovered nearly one hundred years ago. Within four years of the reported discovery and introduction, Sinton and colleagues working in Northern India observed that the combination of quinine and plasmoquine was effective in preventing late recurrences (presumed relapses) of *Plasmodium vivax* malaria (1,2). This efficacy against late attacks of vivax malaria was very slow to be accepted by international authorities (notably the malaria commission of the League of Nations) and malaria experts. Plasmoquine was not well tolerated at the doses required for radical cure: abdominal pain and vomiting were dose limiting, and “cyanosis” was noted with higher doses (3–5). It was also observed that in patients of African or Asian origin, about 10% of developed significant haemolytic anaemia (5). The cyanosis following pamaquine therapy was caused by methaemoglobinaemia, and the sporadic severe haemolytic anaemia was later identified as resulting from the oxidant drug susceptibility of erythrocytes with reduced glucose-6-phosphate dehydrogenase (G6PD) activity. During the Second World War recurrent vivax malaria in the Indo-Burman and Pacific theatres of war was a major threat to soldiers on both sides. An intensive research effort, based in the United States, set out to discover more effective and better tolerated 8-aminoquinolines. Pharmacometric studies in volunteers infected with *P. vivax* (notably the frequent relapse Chesson strain originating in New Guinea) and in rhesus monkeys infected with *P. cynomolgi* continued after the end of the Second World War, and ultimately led to the replacement of pamaquine by primaquine in 1951 during the Korean war (6). These large and detailed pioneering volunteer studies recorded a wealth of valuable information.

### Relapse in experimental and naturally acquired vivax malaria

In the experimental malaria studies, non-immune volunteers were infected either by blood taken from a malaria patient or by infected mosquito bites. The volunteers were then observed daily, usually for at least one year (many were prison volunteers). Building on the extensive experiences of malariatherapy of neurosyphilis during the early to mid-20^th^ century, which had refined and standardized the methods of malaria infection and clinical management, at least five and usually ten infected (i.e. sporozoite positive) laboratory reared anopheline mosquitoes were used to initiate the infection. This ensured a substantial sporozoite inoculum was delivered to the recipient. With the Chesson strain of *P. vivax* the consequent early relapse rate was 100% (7).

This high relapse rate in experimental malaria should be contrasted with natural infections in which a single mosquito, with an infection of variable age (and thus transmission potential), infects an individual who may already have significant natural immunity which attenuates illness (8). This distinction is important because it has a substantial bearing on the binary outcome of the therapeutic response to 8-aminoquinolines i.e. relapse or no relapse. Very high relapse rates usually result from multiple inoculations (e.g. in soldiers fighting in the Pacific theatre of World War 2) (9). Overall, the relapse prevention (i.e. radical curative) efficacy of 8-aminoquinolines was substantially better in clinical practice compared with the stern test applied in the volunteer infections. For example, the combination of quinine and plasmoquine at doses ≥ 30mg base/day was considered highly effective in preventing relapse in clinical studies in endemic areas, but doses below 90mg base/day were relatively ineffective in the experimental challenge model (2, 3, 10, 11). Thus, to compare radical curative efficacies it is necessary to consider both drug dosage, and thus drug exposure, and also the likely hypnozoite burden (12).

### Predictors of relapse

Several different factors determine whether a *P. vivax* or *P. ovale* malaria infection will relapse. The hypnozoite burden, geographic origin (i.e. “strain”), and degree of immunity are all important determinants. Liver stage immunity may affect hepatic schizont development, and blood stage immunity suppresses the relapse parasitaemia to an extent that it can be asymptomatic, subpatent, or both (13, 14). Although there is no evidence of acquired 8-aminoquinoline resistance in hypnozoites, parasites do differ in intrinsic susceptibilities. For example, the long latency *P. vivax* “strains” prevalent in temperate regions are more susceptible to 8-aminoquinolines than tropical frequent relapse strains, and *P. vivax* parasites in Southeast Asia and Oceania appear to be less susceptible (i.e. require a larger primaquine dosage) than other tropical “strains” (15).

From a therapeutic perspective exposure to the biologically active oxidant metabolites of the 8-aminoquinoline is the critical factor determining relapse prevention (radical cure) efficacy (16, 17). The same metabolites are thought to cause haemolysis in G6PD deficiency. This exposure results from the total dose absorbed, and the degree of biotransformation to the active metabolites. Patients with cytochrome P450 2D6 polymorphisms which confer reduced function have reduced production of primaquine’s bioactive metabolite(s) and correspondingly reduced radical curative efficacy (18,19). The oxidative activity of the 8-aminoquinoline treatment is also reflected in the intraerythrocytic concentrations of methaemoglobin. The association of methaemoglobinaemia with radical curative efficacy of 8-aminoquinolines is discussed here.

### Methaemoglobinaemia

**Figure 1:**
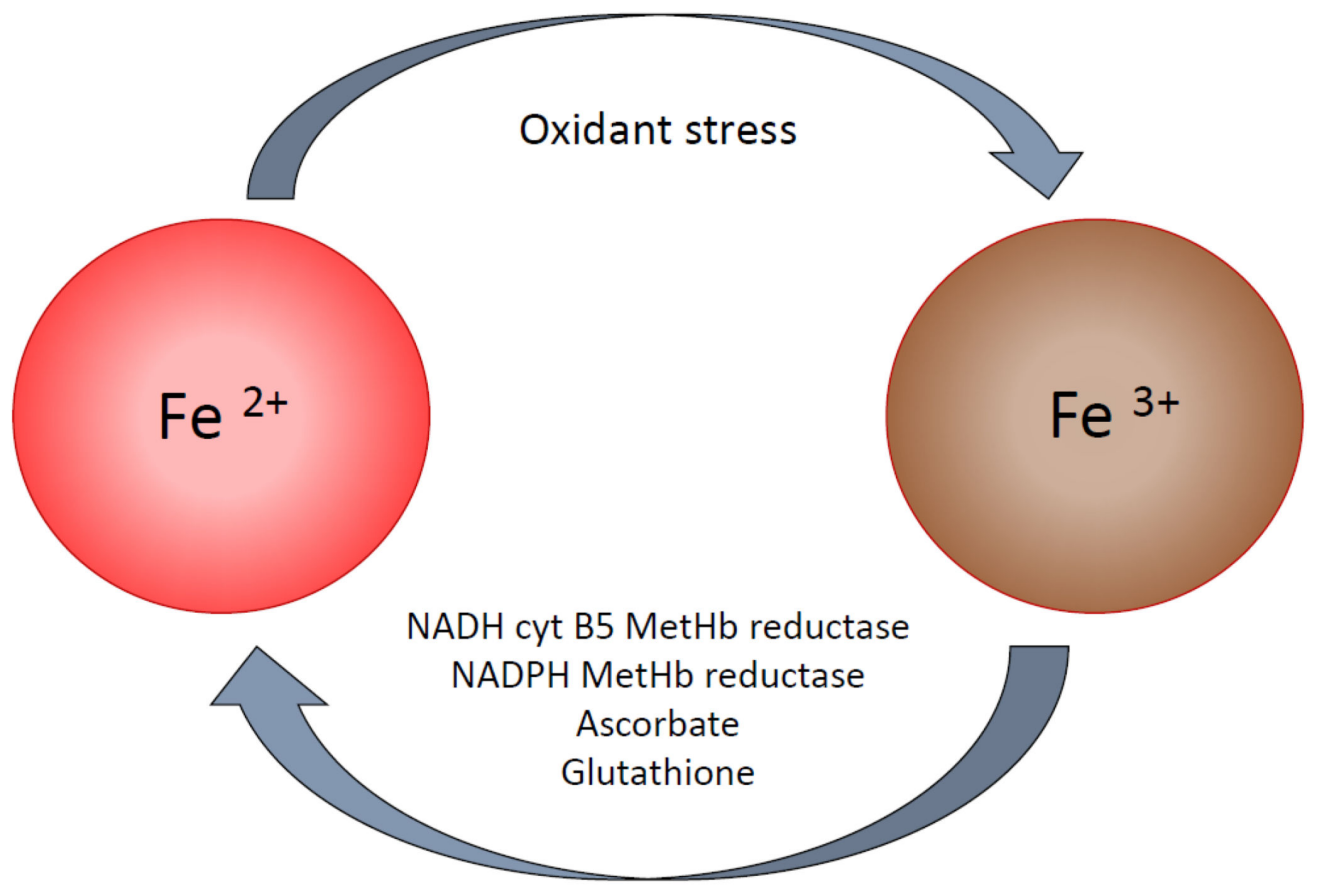
Intraerythrocytic haemoglobin - methaemoglobin interconversion. Each day approximately 3% of the red cells’ haemoglobin is oxidized to methaemoglobin. The major factor contributing to reduction of methaemoglobin back to haemoglobin is the activity of the red cell NADH cytochrome B5 MetHb reductase. MetHb: methaemoglobin.

Methaemoglobin results when the haem iron in haemoglobin is converted from the ferrous state (Fe^2+^), the reduced form, to the (Fe^3+^) ferric ion, the oxidized form (20). This process is coupled to redox cycles in the red cell. In the main cycle, driven by the NAD-cytochrome b5 reductase (the main methaemoglobin reductase), haemoglobin and methaemoglobin are cycled (Figure 1). In the second, a cell redox cycle system is driven by the oxidation of haemoglobin, with methaemoglobin as the product. Although the main enzyme responsible for intraerythrocytic methaemoglobin reduction is the NADH-cytochrome b5-reductase, there are alternative pathways. These include an NADPH-dependent methaemoglobin reductase (which has substantially reduced activity in G6PD deficiency), and direct reduction by intracellular ascorbate and glutathione (Figure 1). Methaemoglobin reduction is a first order process. Under normal steady state conditions approximately 3% of the body’s haemoglobin is oxidized each day to methaemoglobin but, because of back-conversion, the average proportion of methaemoglobin is less than 2% of the corresponding haemoglobin concentration. Numerous factors influence this balance (including foods, drugs, exercise, smoking, hypoxia).

**Figure 2:**
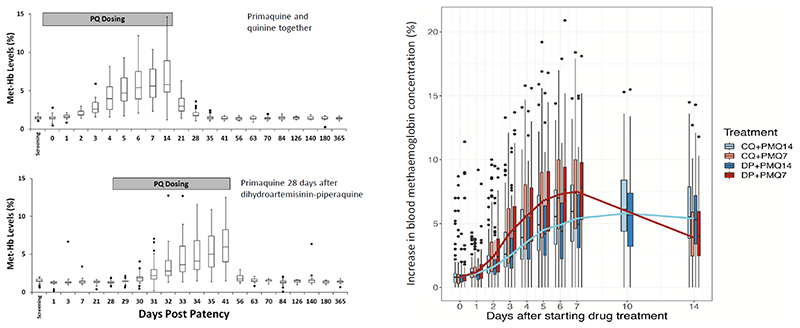
The figures show increasing levels of methaemoglobinaemia (as a proportion of the corresponding haemoglobin concentration) during radical curative treatment of vivax malaria with primaquine. On the left (Figure 2A) primaquine 30mg base/day was given to adults for 14 days either concurrently with quinine, or 28 days after a treatment dose of dihydroartemisinin-piperaquine (from Sutanto et al (21)). On the right (Figure 2B) primaquine was given to adults and children either at a dose of 0.5mg base/kg/day for 14 days or 1mg/kg/day for 7 days and patients were randomized to receive concurrent chloroquine or dihydroartemisinin-piperaquine (from Chu et al (22))

Methaemoglobin is a dark blue-brown (“chocolate”) colour compared with the bright red of oxygenated haemoglobin, so the skin colouration in methaemoglobinaemia resembles that in cyanosis caused by increased concentrations of deoxygenated haemoglobin. Methaemoglobin has a distinct spectrum from haemoglobin. Methaemoglobinaemia can therefore be measured by spectrophotometry of fresh blood samples in the laboratory or, utilizing the same principle, by continuous transcutaneous oximetry devices. During exposure to the oxidizing agent methaemoglobinaemia increases, although there is substantial interindividual variation in iatrogenic methaemoglobinaemia with a skew distribution of steady state values. Interquartile ranges typically extend from approximately 60 to 140% of the median values and, for a given drug exposure, individual values can range tenfold in large series. With daily primaquine dosing methaemoglobinaemia has an estimated elimination half-life of approximately 1.5 days (Figure 2) i.e. 90% of the new steady state is reached in approximately 6 days.

**Figure 3:**
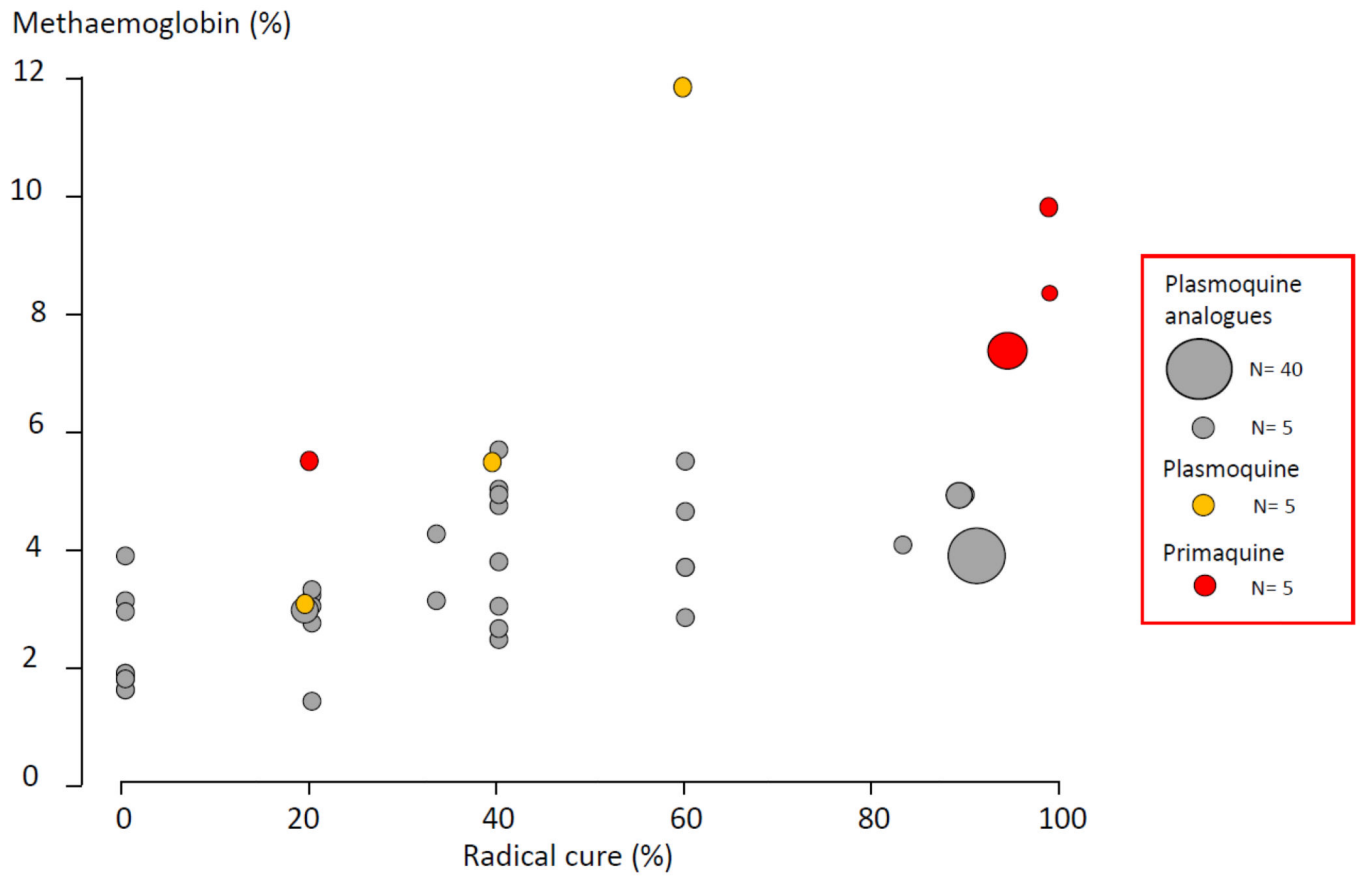
During the clinical investigations conducted over 70 years ago in the USA to develop new 8-aminoquinolines 18 different compounds were evaluated in addition to pamaquine, and later primaquine (23–25). Each was administered for 14 days concurrently with quinine. The volunteers were infected by multiple bites of *A. quadrimaculatus* infected with the Chesson strain of *P. vivax*. The figure shows the relationship between the radical cure proportion and the average blood methaemoglobin concentration (%) measured in the last 4 days of treatment. The size of the circles is proportional to the number of subjects recruited as shown in the inset box.

Several different oxidant chemicals and drugs can cause methaemoglobinaemia. Although methaemoglobin production (i.e. haem oxidation) by 8-aminoquinolines is generally considered not to lie in the causal pathway to haemolysis or antimalarial activity (26), it does provide an approximate correlate of these activities within the 8-aminoquinoline class. Under the rigorous test of the experimental *P. vivax* challenge studies conducted in the USA in the late 1940s and early 1950s, drugs or drug concentrations which produced less than 6% steady state methaemoglobinaemia were associated with sub-optimal radical cure rates (23–25) (Figure 3). As for iatrogenic haemolysis, there is a slight delay before intraerythrocytic methaemoglobin levels begin to rise following 8-aminoquinoline administration. This presumably reflects depletion of the intrerythrocytic oxidant defences (notably reduced glutathione) (27). In a large recent study of vivax malaria, conducted on the Thailand-Myanmar border, the relationships between primaquine and carboxyprimaquine plasma concentrations, relapses, CYP2D6 polymorphisms and methaemoglobinaemia were investigated. After adjusting for age and partner drug, the day 7 concentrations of primaquine and carboxyprimaquine were not associated with the risk of recurrence, but methaemoglobinaemia was. Higher levels of methaemoglobinaemia were associated with reduced recurrences; a 1% absolute increase in day 7 methemoglobin was associated with a hazard ratio for recurrence of 0.9 (95% CI: 0.85-0.99, p=0.02) (28).

**Figure 4:**
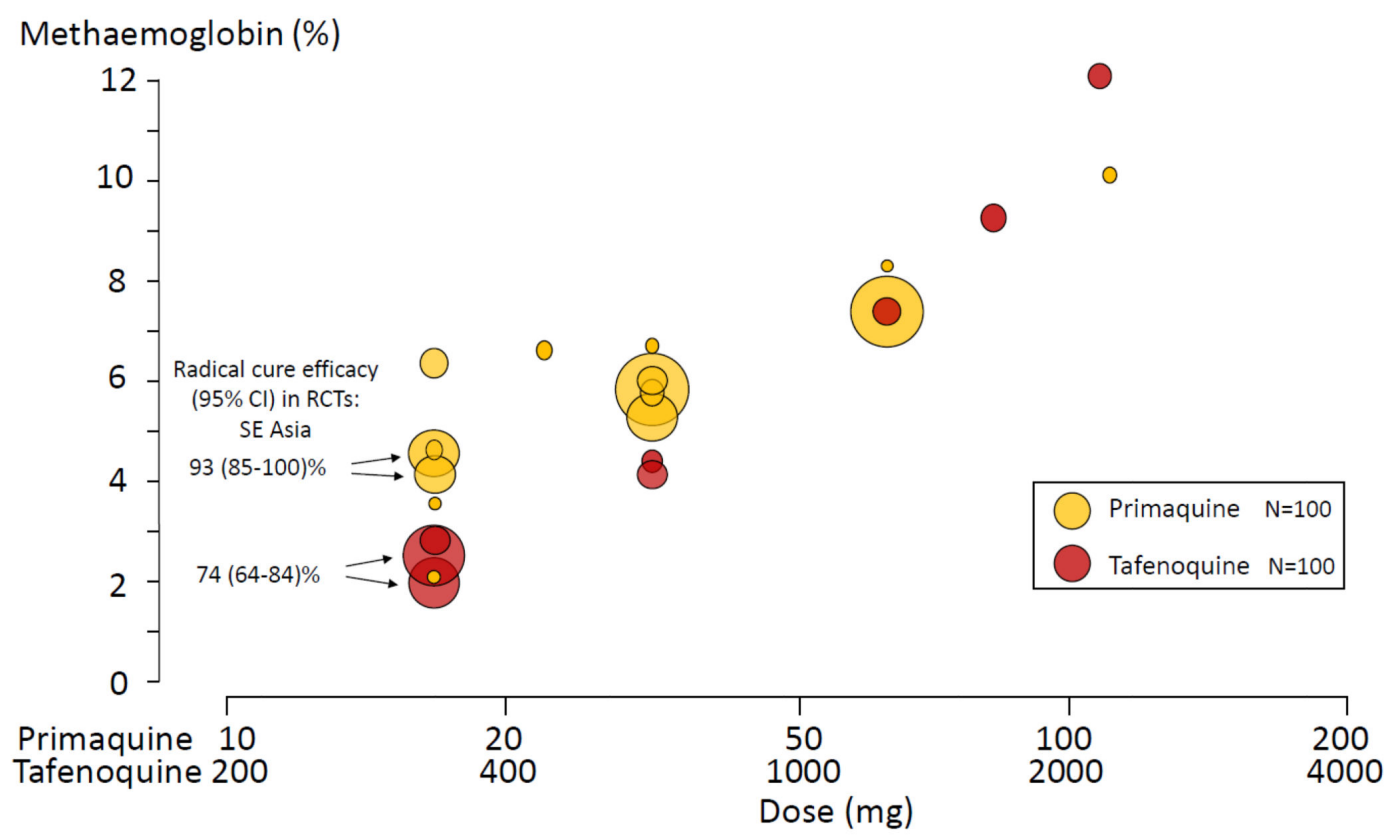
Dose-response relationships for primaquine and tafenoquine in generating methaemoglobinaemia (21,22, 24–26, 28–32). Peak values are shown. The dose shown is the daily adult dose of primaquine, whereas for tafenoquine the dose shown is the total dose administered. Primaquine was administered with chloroquine or quinine, tafenoquine was administered with chloroquine or alone. The radical cure rates in Southeast Asia in the two large randomized trials (29,42) are shown, in which the adult primaquine dose was 15mg base/day for two weeks versus a single 300mg dose of tafenoquine. The corresponding average peak methaemoglobin levels from these two trials are indicated by arrows. The size of the circles is proportional to the number of patients or volunteers recruited as shown in the inset box.

Tafenoquine is a slowly eliminated 8-aminoquinoline which has been introduced recently. At the currently recommended dose (adult dose 300mg) tafenoquine provides radical cure rates which are significantly inferior to the lower dose of primaquine (total dose; 3.5mg/kg) in South-East Asia (29). It is notable that this tafenoquine dose is associated with methaemoglobin concentrations which approximately half those associated with the lower dose primaquine regimen (30,31) and one third of those associated with the currently recommended primaquine radical cure regimen (total dose; 7mg/kg) in the region (Figure 4).

### Drug interactions

**Figure 5:**
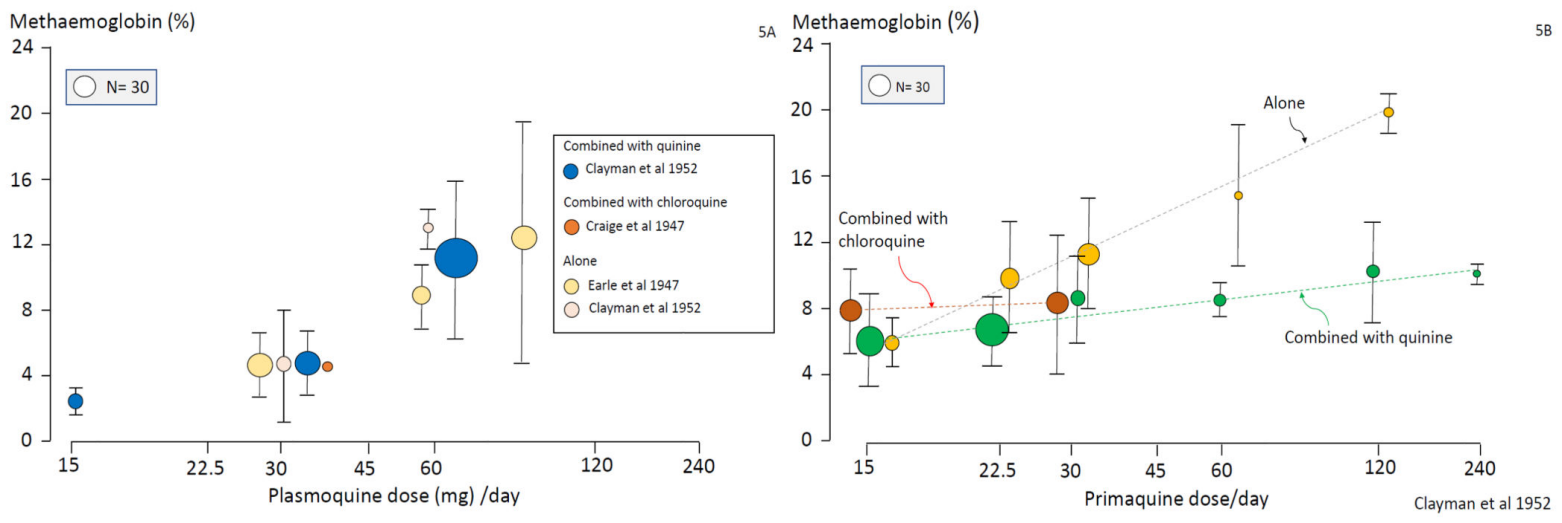
Studies conducted during the development of primaquine which assessed the average methaemoglobin concentrations (%) as a proportion of the haemoglobin concentrations in the last 4 days of treatment in relation to the dosing of plasmoquine (pamaquine) (left) and primaquine (right) (11,33,37). These initial observations suggested that methaemoglobinaemia following primaquine, but not plasmoquine, was attenuated by concomitant administration of chloroquine or quinine. However, later studies (summarized in Figure 6) did not confirm this. The size of the circles is proportional to the number of patients or volunteers recruited as shown in the inset box.

**Figure 6:**
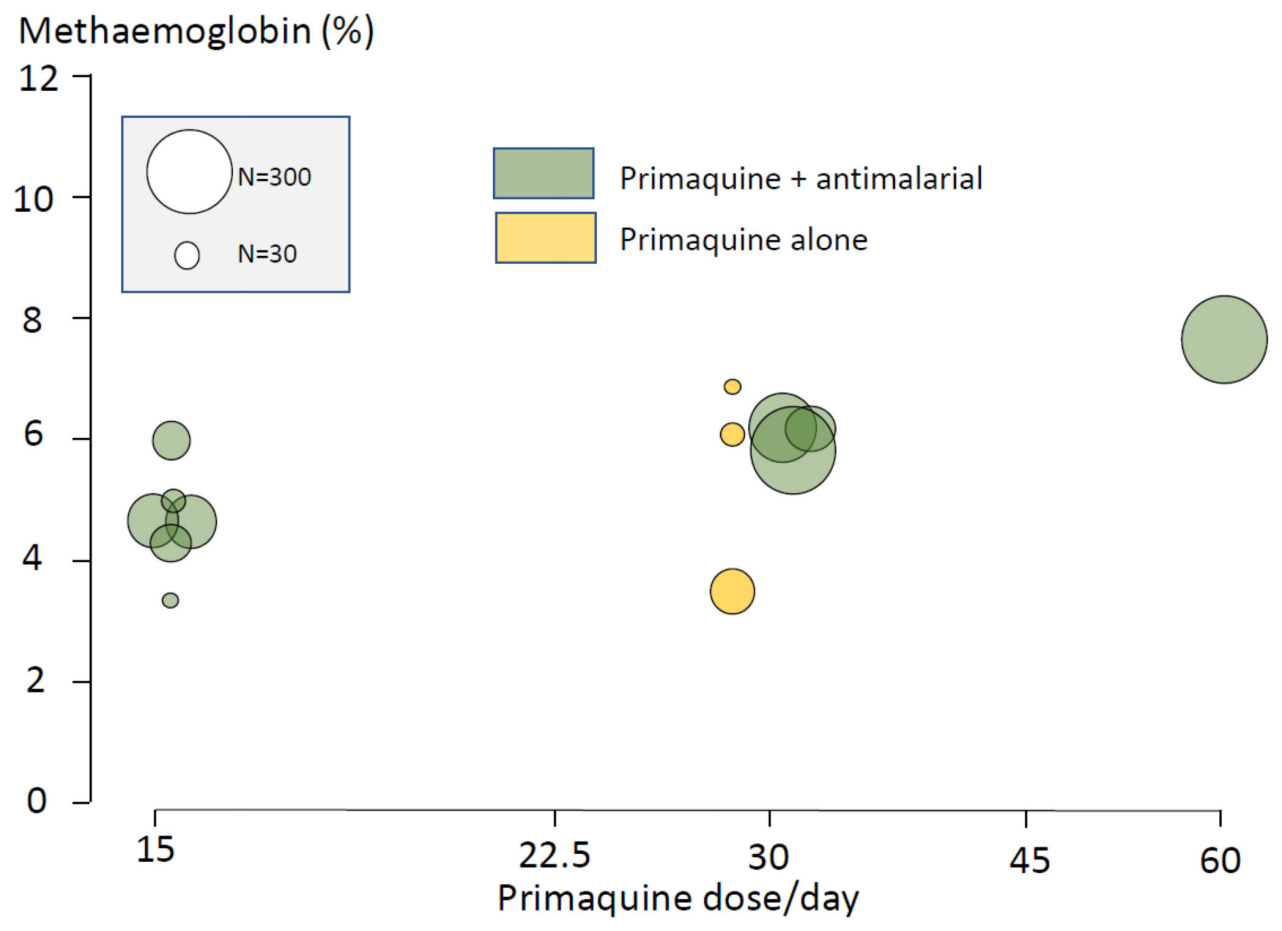
More recent studies in which methaemoglobinaemia has been assessed after different dose regimens of primaquine whether given alone (26,39–41), or in combination with antimalarial drugs (18, 34, 37, 38), either to healthy volunteers, in antimalarial prophylaxis, or in the treatment of vivax malaria (15,19,21,22,24–26,28–32,37,42,43). These do not suggest attenuation of primaquine induced methaemoglobinaemia by concomitant antimalarial drugs (as reported by Clayman et al (37)), nor are there differences between healthy subjects and patients with malaria. The size of the circles is proportional to the number of patients or volunteers recruited, as shown in the inset box.

The 8-aminoquinolines have important pharmacokinetic and pharmacodynamic interactions with other structurally related antimalarial drugs. Coadministration of mepacrine (atebrin, quinacrine) substantially increases plasma concentrations of pamaquine and also reduces tolerability. It also substantially increases the methaemoglobinaemia associated with pamaquine (33). Chloroquine, piperaquine and pyronaridine all increase plasma concentrations of primaquine by approximately 20%, but they do not affect tolerability (34). Pamaquine and primaquine radical curative activity is increased by concomitant (but not sequential) administration with quinine (35, 36). Chloroquine has also been shown to potentiate the radical curative efficacy of primaquine. During the clinical development of primaquine it was observed that coadministration of primaquine with quinine or chloroquine attenuated the consequent methaemoglobinaemia. This was not observed in comparable studies with pamaquine (plasmoquine) (Figure 5). However larger, more recent, studies have not confirmed this early finding (Figure 6). They do not suggest any substantial differences between methaemoglobin levels with or without concomitant medicines (quinine, chloroquine, artesunate, artesunate-pyronaridine, and dihydroartemisinin-piperaquine have all been evaluated), nor do they suggest differences between methemoglobin levels in the treatment of malaria (in which steady state methaemoglobin levels are reached well after resolution of symptoms), or in healthy subjects. Thus the impact of blood schizontocides on 8-aminoquinoline induced methaemoglobinaemia is specific to the drugs and is not a general class effect.

### Methaemoglobinaemia and G6PD deficiency

**Figure 7:**
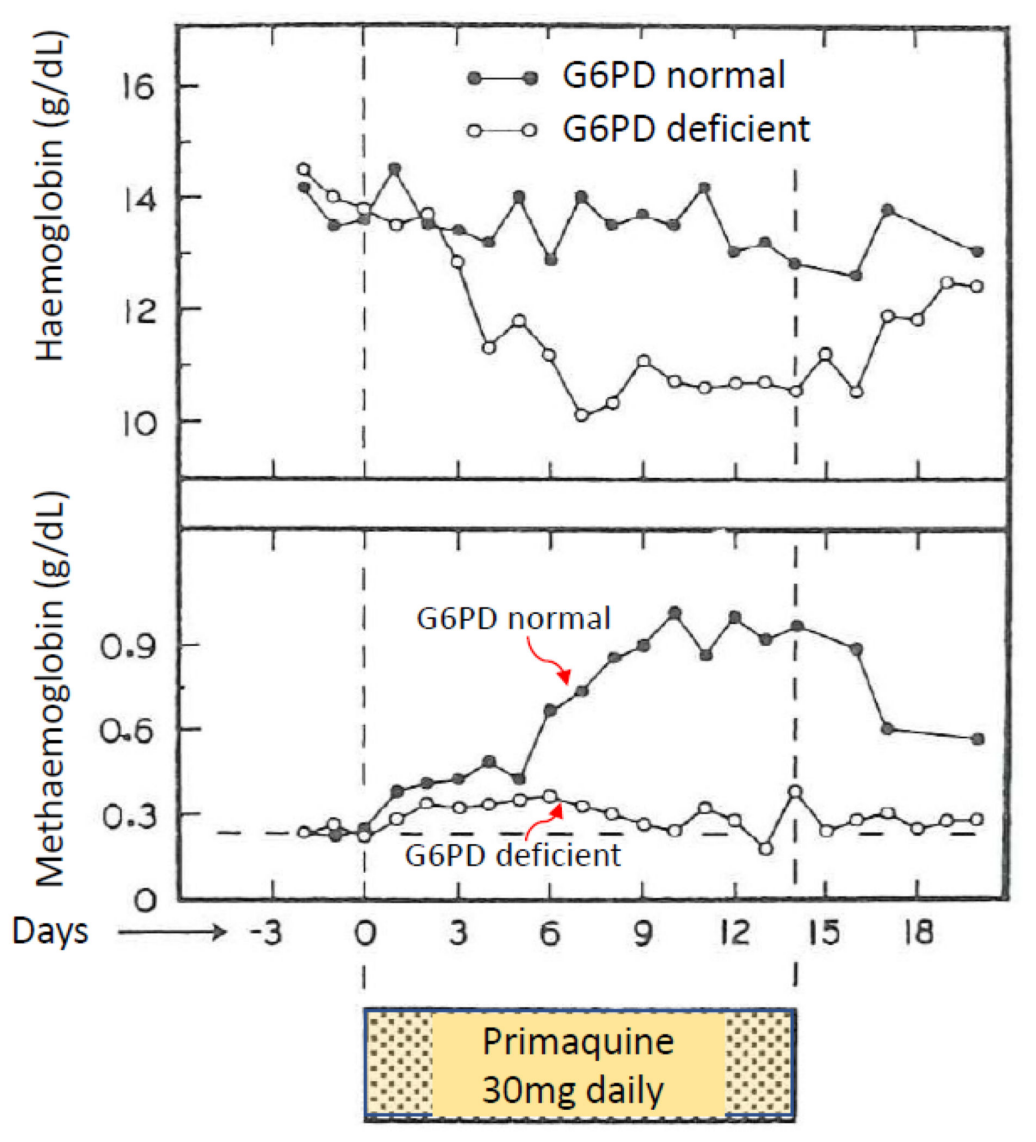
Mean values of haemoglobin (g/dL) and methaemoglobin (%) in 12 normal and 12 African A-G6PD deficient adult volunteers given primaquine (from Brewer et al (26)).

The discrepancy between 8-aminoquinoline haemolytic toxicity and methaemoglobinemia has been noted widely (26). In the research which led to registration of primaquine, Edgcomb et al (24) noted no correlation between haemolysis and methaemoglobinaemia following pamaquine or primaquine. The erythrocytes in G6PD deficiency have a reduced ability to reduce methaemoglobin in the presence of “electron-donors”, such as the bioactive metabolites of primaquine or methylene blue (this is the basis of the methylene blue methaemoglobin reduction test developed to diagnose G6PD deficiency). This is because of the reduced intraerythrocytic activity of the NADPH dependent methemoglobin reductase. Brewer et al showed that whereas G6PD deficient individuals had *increased* levels of methaemoglobinaemia following oral sodium nitrite (an oxidizing agent which does not cause haemolysis), they had *decreased* levels of methaemoglobinaemia following primaquine (26) (Figure 7). This apparent paradox was explained by the iatrogenic haemolysis of the older erythrocytes which contained the highest concentrations of methaemoglobin. Other mechanisms are also possible, such as the sequestration of the primaquine oxidant metabolites in the oxidized haemoglobin Heinz bodies. The mechanism of 8-aminoquinoline haemolytic toxicity has not been explained satisfactorily and, although increased oxidant stress is implicated, there are clearly other processes also involved.

### Conclusions

Methaemoglobinaemia is associated with exposure to the biologically active metabolites produced by therapeutic doses of 8-aminoquinoline drugs. The proportion of methaemoglobin to haemoglobin in blood correlates with the efficacy of anti-relapse therapy, but methaemoglobin appears to be uninvolved directly either in that activity or in haemolytic toxicity in G6PD-deficient patients. This suggests that the oxidative processes which result in methaemoglobinaemia are necessary, but they are not sufficient, for the radical curative activity of the 8-aminoquinoline antimalarial drugs. Although there is substantial inter-individual variation in iatrogenic methaemoglobinaemia, the overall clinical data suggest that methaemoglobinaemia is a pharmacodynamic correlate of radical curative activity. Measurement of methaemoglobinaemia might be useful in drug screening and dose evaluation as a prelude to definitive phase 3 studies characterizing safety and efficacy

## Data Availability

All data used are available from the original publications.

## • What is Already Known About This Subject

The 8-aminoquinoline antimalarials are the only drugs which can prevent relapses of *P. vivax* and *P. ovale* malaria (radical cure), but no pharmacodynamic correlates of this activity have been identified. The 8-aminoquinolines cause dose dependent methaemoglobinaemia, and they cause haemolysis in patients with G6PD deficiency.

## • What This Study Adds

Review of data accrued over the past 70 years indicates that methaemoglobinaemia is correlated with radical curative activity. Methaemoglobinaemia could be used to optimize 8-aminoquinoline antimalarial dose regimens.
